# Activity and Cognition in Older Adults With and Without Sleep Apnea

**DOI:** 10.1093/geroni/igaa057.709

**Published:** 2020-12-16

**Authors:** Nora Mattek, Jeffrey Kaye, Zachary Beattie

**Affiliations:** 1 Oregon Health & Science University, Portland, Oregon, United States; 2 Layton Alzheimer’s Disease Center, Portland, Oregon, United States

## Abstract

Many older adults have sleep apnea. It often goes undiagnosed. Sleep apnea is associated with decreased oxygen to the brain, fragmented sleep, shorter sleep duration, impaired cognition, increased risk for dementia, and less clearance of amyloid and tau. Little longitudinal data are available identifying objective measures of sleep apnea over time that may facilitate improved identification of affected persons. One hundred ninety-five participants age 57 or older were enrolled as part of Collaborative Aging Research using Technology (CART) initiative, a national study examining the feasibility of unobtrusive remote sensing and monitoring of cognitive, behavioral, physiological, and health-related activities. Nightly hours of sleep and total daily step count were acquired using a wrist worn device. Sixty (31%) reported having sleep apnea. Those with sleep apnea had a significantly lower MoCA total score than those without (22.9 vs. 24.2, p<0.01) and were more likely to be classified as MCI by MoCA cutoff <23 (49% vs. 24%, p<0.001). Volunteers who live alone were less likely to be diagnosed with sleep apnea (p<0.01). Males were much more likely to report having sleep apnea than females (p<0.0001). Of those with watch-derived activity data, sleep apnea cases had shorter mean sleep duration (7.2 vs. 7.8 hours, p=0.05) and fewer total daily steps than those without sleep apnea (2384 vs 3327, p=0.04). Sleep apnea may be identified by its association with mildly impaired cognition, with shorter sleep duration, and less total daily activity as measured at home via remote continuous monitoring techniques.

